# Regulating the product quality of COVID-19 antigen testing reagents: A tripartite evolutionary game analysis under China's legal framework

**DOI:** 10.3389/fpubh.2022.1060079

**Published:** 2023-01-09

**Authors:** Zhengzong Huang, Xi Wang, Zehua Feng, Baoxin Chen

**Affiliations:** ^1^Law School, Hainan University, Haikou, China; ^2^Faculty of Humanities and Social Sciences, Macao Polytechnic University, Macao, China; ^3^School of Law, Guangdong University of Technology, Guangzhou, China

**Keywords:** regulatory authorities, inspection agencies, law enforcement, medical device, false testing results

## Abstract

Personal purchases of novel coronavirus antigen detection reagents (ADRs) for self-detection have contributed to the optimization of medical resources and containment of the COVID-19 pandemic. The recurring occurrence of false testing results in China has generated concerns regarding the quality of ADRs and the testing mechanism for medical devices. Academic viewpoints and remarks on the sensitivity, application possibilities, and product innovation of ADRs may be found in the extant scientific literature. However, the current research does not explore the microscopic product quality concerns that emerge throughout the production and marketing of ADRs. To explore strategic equilibrium circumstances and behavioral evolution processes, an evolutionary game model was developed to include ADR manufacturers, third-party medical device inspection agencies, and regulatory authorities. The results reveal that the quantity of illegal incentives, the cost of regulation, and the loss of government credibility have a major impact on the decisions of regulatory authorities and determine three potential systemic equilibrium states. To maximize social welfare, ADRs should be incorporated into China's medication price monitoring system in order to manage market prices. To cut regulatory expenses, the government should employ blockchain technology for traceable network regulation of ADR product quality. The government should also protect the people's right to free speech and encourage online reporting of adverse incidents caused by ADRs. The conclusions of this article can provide many developing nations with important insights for regulating the quality of ADR products.

## 1. Introduction

The coronavirus outbreak in 2019—officially the COVID-19 pandemic—still remains the most serious global public safety incident in recent years (1). COVID-19 has seriously affected public health, economic development, social stability, and even national security worldwide (2). Because of its extensive spread, this disease emerged as an urgent worldwide concern to citizens and governments alike (3). With the gradual decline in the severity and fatality rates of COVID-19 in combination with increased vaccination rates, some countries and regions have begun to relax their COVID-19 prevention and control measures (4, 5). On March 11, 2022, the Chinese government issued the *Application of the COVID-19 Antigen Detection Program* (6) to further implement its “dynamic zero-COVID-19” policy (7). The program allows anyone to obtain COVID-19 antigen detection reagents (ADRs) for self-testing in order to enhance early identification and treatment of COVID-19 (8). The China National Medical Products Administration (NMPA) had authorized 31 COVID-19 ADR products in accordance with government regulatory processes as early as April 29, 2022 (9). These ADRs are already available for purchase. Guangzhou, the capital of China's Guangdong Province, removed its city lockdown on December 1, 2022 in the face of a major COVID-19 infection, marking a turning point in the change of China's pandemic prevention policy (10). The government of Guangzhou supplies the public with free ADRs and encourages them to purchase ADRs for self-testing. ADRs will be mass-produced, sold, and utilized in China.

ADRs are classified as Class III medical devices under China's Regulation on the Supervision and Administration of Medical Devices (hereinafter Medical Devices Regulation). Class III medical devices pose a greater risk, necessitating stricter management controls to guarantee their safety and efficacy. No one is permitted to engage in the production or commercialization of Class III medical devices without authorization. The government should oversee medical device research, production, operation, and use. Third-party medical device inspection agencies (hereinafter “inspection agencies”) can provide quality certification services for the government and manufacturers of ADRs (11). They have become vital evaluators of quality certifications for medical devices being brought to the market (11). To develop high-quality products, medical device firms must pay substantial research and production expenses (12). Conversely, low-quality products normally have low production costs and large profit margins (12). Hence, ADR manufacturers frequently have a temptation to bribe inspection agencies (13, 14). This situation is difficult to control. In the presence of limited government supervision, ADR manufacturers tend to produce low-quality products and seek leniency from inspection agencies when obtaining marketing licenses. Lack of government monitoring and medical device inspection may lead to low-quality ADRs that have not undergone rigorous medical-standard manufacture (15). Low-quality ADRs sold to customers as a result of unethical business tactics can lead to an abundance of erroneous diagnostic results (i.e., false negative results and false positive results). As a result, those who self-test using substandard testing kits do not realize that their results may be misleading. Inaccurate self-testing results could cause SARS-CoV-2 to spread wider across the nation (16, 17).

China has experienced the above-mentioned problems. Falsified COVID-19 nucleic acid detection results hurt pandemic prevention efforts. In April 2022, some medical labs issued false testing results on numerous occasions, which has interfered with the overall pandemic prevention and control initiative (18). In May 2022, a testing center issued false positives to 13 residents in Shanghai within one day, but in the subsequent nucleic acid review, all tests were negative (19). These successive occurrences of false testing events are closely related to the profiteering aspect of nucleic acid detection work. Since the outbreak of the COVID-19 pandemic, statistics verify that several of these novel coronavirus detection reagent companies have achieved substantial revenue growth. The net profit of these institutions in the first quarter of 2022 reached 14.3 billion yuan, equivalent to a daily net profit of 158 million yuan (20). According to estimates by the Soochow Securities Research Institute, if all first- and second-tier cities in China (representing a total population of 505 million in 2021) were to implement normalized nucleic acid detection in the future, the monthly cost will be capped at 121.2 billion yuan; this total—equivalent to 1.45 trillion yuan per year—would equal 1.27% of China's nominal GDP in 2021 (21). Given the massive revenue-generating potential, some ADR manufacturers may purposely make low-quality ADRs that decrease the accuracy of nucleic acid detection kits.

Academic research into the regulation of ADR product quality has significant implications for public health (22). To date, however, the academic community has failed to fully addressed the problem of low-quality ADRs and erroneous diagnostic results. In an effort to resolve this problem, the authors of this paper employ evolutionary game theory. First, our examination and analysis of the current literature reveals that researchers have focused solely on the accuracy, potential applications and product advances of antigen detection, while ignoring the product quality of ADRs. Second, we design a tripartite evolutionary game model with ADR manufacturers, inspection agencies, and regulatory authorities as the primary entities and assess the strategic stability of each player and the impact of each variable on their best strategic decisions. Third, a simulation analysis is performed using MATLAB 2021a to validate the model's validity and sensitivity under various scenarios. Lastly, based on the findings, we provide countermeasures and recommendations to enhance the quality of ADR in China from a legislative and enforcement standpoint. This paper's discussion may serve as a resource for other developing nations pursuing similar answers.

## 2. Literature review

Recent research on ADRs has focused on three major areas: the accuracy of antigen detection, the potential applications of ADRs, and product advancements for SARS-CoV-2 antigen detection kits.

Numerous academics have demonstrated the accuracy of antigen detection. In periods of high viral load, Zhan et al. found that reverse transcriptase polymerase chain reaction (RT-PCR) testing and rapid antigen test (RAT) provided equivalent results, with RAT showing a high level of sensitivity when utilized for continuous screening (23). Aoki et al. investigated the Espline(R) SARS-CoV-2 antigen test. They discovered that the antigen test could identify high-risk groups with high virus loads, potentially preventing a pandemic (24). Favresse et al. proposed that sensitive detection of Nucleocapsid-antigen (N-antigen) in serum might assist in identifying individuals at risk of developing severe COVID-19 and, therefore, better optimize intensive care consumption (25). Gupta et al. stated that antigen-based rapid diagnostic tests (RDTs) with high specificity and moderate sensitivity might aid in the quick identification, isolation, and treatment of COVID-19 patients (26). Hober et al. discovered that serologic assays based on several SARS-CoV-2 antigen combinations provide particular and sensitive multiplex serologic testing for COVID-19 (27). Beck et al. found that antibody microarray assays have clinical sensitivity and specificity equivalent to FDA-approved antigen testing (28). According to Grant et al., antigen-based SARS-CoV-2 tests may offer higher clinical sensitivity than serological assays (29).

Several researchers concentrated on the potential applications of ADRs. Lv et al. reviewed the methodologies and requirements for assessing the clinical performance of quick diagnostic tests for SARS-CoV-2 antigen detection and intended application scenarios of ADRs (30). Hauser et al. found that the LIAISON SARS-CoV-2 antigen test had excellent specificity but low sensitivity and should only be used for preliminary screening (31). Candel et al. observed that major health organizations, such as WHO and the Ministry of Health of Spain, support employing antigenic testing in various pandemic response methods (32). Williams et al. discovered that using plant platforms producing recombinant proteins as COVID-19 diagnostic reagents might reduce the cost of diagnostic kits (33). According to Varotto-Boccazzi et al., *L. tarentolae* is an efficient method for viral antigen synthesis in low-tech cell factories, with sensitivity and repeatability equivalent to reference antigens generated in human cells (34). Kritikos et al. suggested that RATs might assist in the reduction of the sporadically observed reagent scarcity in RT-PCR tests (35). Shao concluded that while SARS-CoV-2 antigen test findings cannot be utilized to diagnose COVID-19 directly, they may be used for early triage and immediate care of questionable groups. SARS-CoV-2 antigen testing is susceptible to false-positive and false-negative findings due to technical limitations (36). According to Fournier et al., antigen testing is unsatisfactory due to its lack of sensitivity. However, there is a global scarcity of reagents and kits. ADRs have evolved into a necessary alternative (37). Ellipilli et al. claimed that antigen tests could produce findings in <2 h and that diagnostic reagents could be purchased for <$5.00. There are currently commercially accessible lateral flow diagnostic kits with immobilized antibodies that enable patients to be diagnosed at home (38). Singh et al. described an inducer-based SARS-CoV-2 salivary antigen test using low-cost reagents ($3.20/visit) and a commercially available glucometer. This technology offers a low-cost, quick, and accurate diagnostic tool for large-scale distributed screening for SARS-CoV-2 infection (39).

Researchers have also looked at product advancements for SARS-CoV-2 antigen detection kits. According to Zhang et al., SARS-CoV-2 fluorescent immunochromatographic test strips can detect the virus quickly, sensitively, and accurately and meet clinical demands for on-site viral testing (40). Ramanujam et al. produced an ultra-fast SARS-CoV-2 detection sensor capable of detecting coronavirus proteins in saliva in <100 milliseconds (41). Nafian et al. examined several approaches based on clustered regularly interspaced short palindromic repeats (CRISPR) and discussed their advantages and disadvantages for point-of-care detection (POCT) of suspected SARS-CoV-2 infection at home or in small clinics (42).

Academic viewpoints and remarks on the sensitivity, application possibilities, and product innovation of ADRs may be found in the extant scientific literature. Most of these researchers feel that ADRs can compensate for deficiencies in nucleic acid detection. Based on antigen detection experimental data, the majority of the literature focuses on the practicability, validity, and dependability of ADRs. However, the current research does not explore the microscopic product quality concerns that emerge throughout the production and marketing of ADRs. Numerous false-positive and false-negative results might come from product quality issues, which are detrimental to the successful execution of public health efforts (e.g., personal distancing). Researchers have observed that the marketing of ADRs involves stakeholders such as government agencies, patients, and manufacturers (32, 43–46).

Nonetheless, it is obvious that researchers have not exhaustively analyzed the behavior patterns of different participants. Evolutionary game theory has not applied to the stakeholders in this situation. Evolutionary games offer significant application potential for simulating the behavioral tactics of various participants (47, 48). From a microscopic vantage point, it is possible to examine the strategic decisions and evolutionary logic of many players in the control of ADR product quality. Evolutionary game theory has been utilized extensively in the disciplines of COVID-19 public health governance (49), free riders in healthcare policy (50), and COVID-19 information disclosure (17).

There is little doubt that the government may strengthen public health governance by providing subsidies and imposing administrative fines to assure the product quality of ADRs and the prompt diagnosis of COVID-19 patients. However, inadequate financial resources may not guarantee sufficient subsidies for ADR manufacturers, and weak enforcement capacities may prevent early administrative sanctions for manufacturers of low-quality ADRs. The wider the product profit margin of ADRs, the greater the probability that ADR manufacturers would bribe inspection agencies to submit fraudulent test results, and the more difficult it would be to halt the manufacturing of low-quality ADRs. This requires further research.

Few researchers have examined the actions and decisions of regulatory authorities, inspection agencies, and ADR manufacturers from the standpoint of ADR product quality. In addition, no researcher has included these three parties in an analytical framework to investigate the effects of their interactions and the resulting societal impacts. Consequently, the following contribution is made by this study to the current body of knowledge: we employ a model based on evolutionary game theory that integrates regulatory authorities, inspection agencies, and ADR manufacturers into the analytical framework. We study the equilibrium results of their mutual activities and the accompanying social repercussions. Using the economic premise that people are rational, the evolutionary game model analyzes the learning and adjustment process underlying the behavioral decisions of regulatory authorities, inspection agencies, and ADR manufacturers.

## 3. Methods

### 3.1. Model description

This paper assumes that the total revenue of COVID-19 ADR manufacturers can be divided into two parts, namely, the income from the sale of ADRs (*E*_*e*_) and government subsidies (*S*_*e*_). The cost of producing high-quality ADRs is *C*_*h*_, and the cost of producing low-quality ADRs is *C*_*l*_ (*E*_*e*_>*C*_*h*_>*C*_*l*_).

When a manufacturer produces low-quality ADRs or an inspection agency have the potential for rent-seeking, the manufacturer must pay a related fee (*K*) to the inspection agency. In order to cooperate with the inspection and sale of low-quality ADRs, manufacturers will need to falsify production data, release false publicity, etc., which incurs speculative costs (*V*_*e*_). When the manufacturer produces a low-quality product and puts it on the market, it may be subject to government fines (*P*_*e*_), and *C*_*h*_>*C*_*l*_+*K*+*V*_*e*_+*P*_*e*_+*S*_*e*_.

Inspection agencies that provide detection services can receive income for the services they performed (*E*_*t*_). They may also obtain government subsidies (*S*_*t*_). If an inspection agency accepts bribes from a manufacturer, it will need to bear the cost (*V*_*t*_) of falsifying test data and issuing false testing results, and may face government fines (*P*_*t*_).

High-quality ADRs can more effectively identify patients infected with COVID-19, protect public health, maintain social order, and help generate social benefits (*E*_*g*_) on behalf of the government. If the government more stringently supervises manufacturers and inspection agencies, it usually bears the associated supervision costs (*C*_*r*_). When manufacturers produce and sell low-quality ADRs after bribing an inspection agency, the country's overall ability to prevent and control the COVID-19 pandemic is weakened along with its public health system and economic development. To protect public health and monitor the medical device industry, the government must allocate funds to increase governance (*G*_*g*_) or perhaps bear a decline in its credibility (*P*_*g*_).

**Figure 1:**
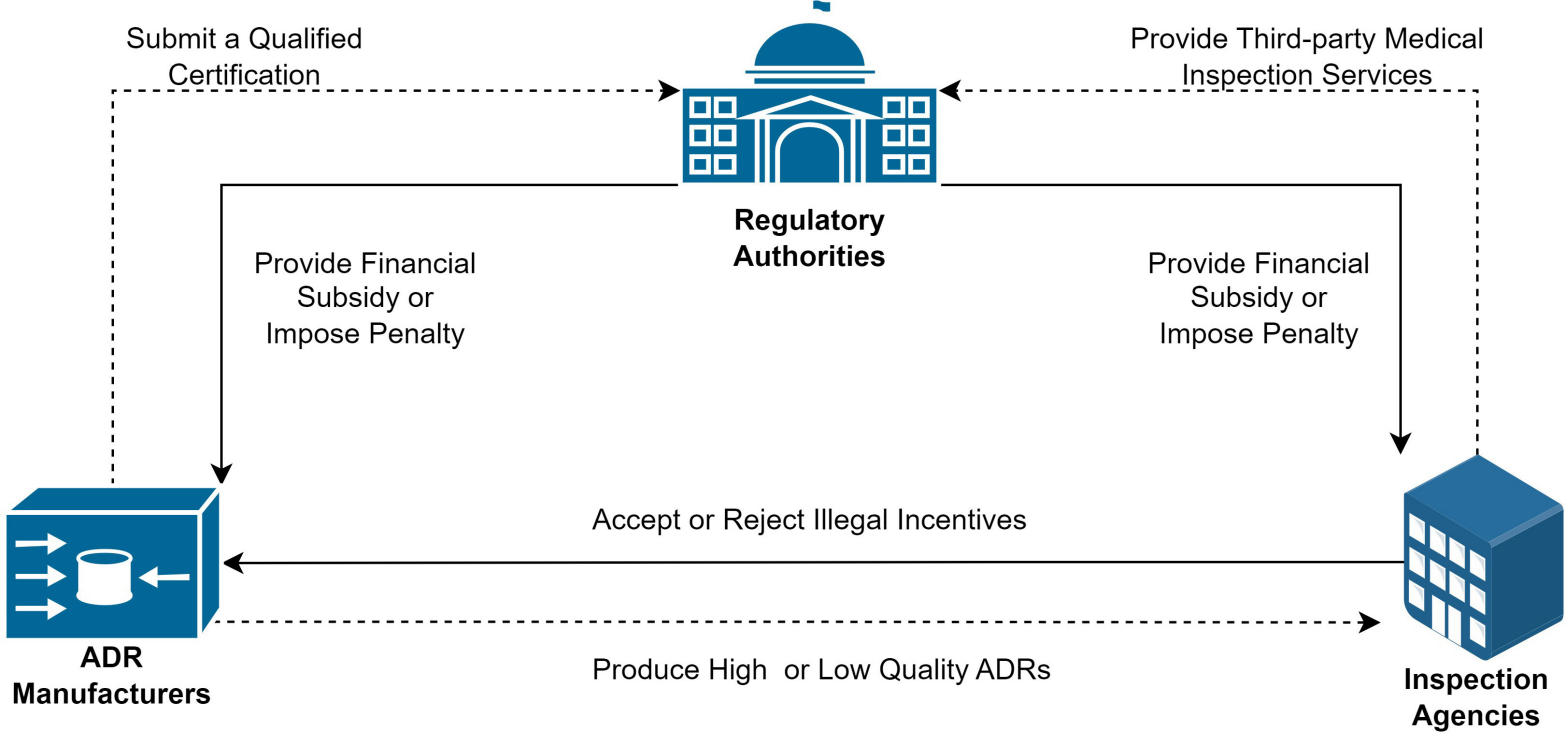
The tripartite evolutionary game model of quality supervision for COVID-19 ADRs.

All variables used in the model and their related definitions are shown in Table 1. The entities and their logical relationships are shown in Figure 1.

**Table 1:** Variables and their definitions.

**Participants**	**Variable**	**Variable definition**
ADR manufacturers	*E*_*e*_	Sales revenue
	*C*_*h*_	Produce high-quality ADRs
	*C*_*l*_	Produce low-quality ADRs
	*K*	Bribes
	*V*_*e*_	Speculative costs
	*P*_*e*_	Government fines
	*S*_*e*_	Government subsidies
Inspection agencies	*E*_*t*_	Operating income
	*V*_*t*_	Speculative costs
	*P*_*t*_	Government fines
	*S*_*t*_	Government subsidies
Regulatory authorities	*E*_*g*_	Social benefits
	*C*_*r*_	Regulatory costs
	*G*_*g*_	Remediation costs
	*P*_*g*_	Government credibility

### 3.2. Assumptions

To construct a viable model, various factors are used to analyze the stability of the strategies and the equilibrium points for each party, as well as the influence of each element, and the following assumptions are made:

Hypothesis 1. Supervising the production of ADRs for 2019-nCoV requires the joint participation of ADR manufacturers, inspection agencies, and regulatory authorities. Enterprises produce ADRs to obtain economic revenue. As profit-seeking entities, it is possible for manufacturers to produce low-quality ADRs to reduce their production costs. In the meantime, manufacturers potentially illegally influence the inspection agencies to obtain marketing licenses more easily. Therefore, regulatory authorities must provide stringent oversight. Medical device production should be supervised strictly, with hefty fines and contract loss for illegally influenced inspection agencies and manufacturers who produce low-quality products. Financial subsidies should be offered to incentivize manufacturers to produce high-quality products and inspection agencies to provide high-quality services.

Hypothesis 2. ADR manufacturers, as private enterprises, normally pursue high profits. Producing high-quality ADRs usually requires higher production costs. Producing low-cost, low-quality ADRs normally incurs administrative penalties from regulatory authorities. Therefore, it is assumed that ADR manufacturers have two strategies: producing high-quality ADRs or producing low-quality ADRs. The probability of manufacturers choosing to produce low-quality ADRs is expressed as *x*, while choosing to produce high-quality ADRs is expressed as (1−*x*). Suppose there are two strategies for inspection agencies: accepting or rejecting illegal incentives. The probability of an inspection agency accepting illegal incentives is *y*, and thus the probability of rejecting illegal incentives is (1−*y*). Government regulatory institutions have two strategies: to pursue strict or loose regulatory standards. The probability of strict supervision by regulatory institutions is *z*, and the probability of loose supervision is (1−*z*).

Hypothesis 3. Each stakeholder is a participant in bounded rationality. Since each model subject seeks to maximize their expected revenue in the presence of information asymmetries, strategy selection gradually evolves over time to converge on the optimal strategy.

Hypothesis 4. If ADR manufacturers produce low-quality products and inspection agencies accept illegal incentives, then regulatory authorities will withdraw government subsidies to ADR manufacturers and inspection agencies and impose administrative penalties, respectively. When regulatory authorities choose a loose regulatory strategy, regulatory authorities will still provide government subsidies but will not punish the production of low-quality products and receipt of illegal incentives.

### 3.3. Payoff matrix

#### 3.3.1. Expected revenue for all parties when supervision is stringent

##### 3.3.1.1. The expected revenue of ADR manufacturers

When ADR manufacturers produce and sell low-quality products, they must bear the production costs (*C*_*l*_) and speculative costs (*V*_*e*_). If an inspection agency accepts illegal incentives, low-quality ADRs may be sold on the market, and the manufacturers obtain income from those sales (*E*_*e*_). However, manufacturers must pay the associated rent-seeking costs (*K*) and government fines (*P*_*e*_). In this scenario, the manufacturer's expected benefit is equal to (*E*_*e*_−*C*_*l*_−*V*_*e*_−*K*−*P*_*e*_).

If an inspection agency refuses the illegal influence, the product will not be sold and the manufacturer will not obtain the proceeds (*E*_*e*_). If the regulatory authorities adopt a strict regulatory strategy, manufacturers must pay fines (*P*_*e*_) levied by the regulatory authorities. In this scenario, the manufacturer's expected benefit is equal to (−*C*_*l*_−*V*_*e*_−*P*_*e*_).

When ADR manufacturers produce high-quality products, they can obtain government subsidies (*S*_*e*_) while bearing the production costs (*C*_*h*_). In the presence of strict government supervision, if an inspection agency asks for illegal incentives, the manufacturer must pay an additional illegal incentive (*K*), but does not need to pay the fine (*P*_*e*_). In this scenario, the manufacturer's expected benefit is equal to (*E*_*e*_−*C*_*h*_+*S*_*e*_−*K*). If an inspection agency does not ask for illegal incentives, the manufacturer's expected benefit is equal to (*E*_*e*_−*C*_*h*_+*S*_*e*_).

##### 3.3.1.2. The expected revenue of inspection agencies

The operating income of inspection agencies is denoted as *E*_*t*_. Regardless of whether ADR manufacturers produce low- or high-quality products, the inspection agencies can obtain government subsidies (*S*_*t*_) from the regulatory authorities as long as they refuse illegal incentives. When an inspection agency refuses the illegal incentives, the expected benefit is equal to (*E*_*t*_+*S*_*t*_). Conversely, they must pay both the speculative cost (*V*_*t*_) and the government fine (*P*_*t*_) if the inspection agencies accept the illegal incentive (*K*). In this case, the inspection agency's expected benefit is equal to (*E*_*t*_−*V*_*t*_−*P*_*t*_+*K*).

##### 3.3.1.3. The expected revenue of regulatory authorities

Strict government regulation requires a certain cost of regulation (*C*_*r*_). When manufacturers produce low-quality ADRs, the regulatory authorities impose fines (*P*_*e*_) on manufacturers. If an inspection agency accepts an illegal incentive, the regulatory authorities impose a fine (*P*_*t*_) on the inspection agency. When manufacturers provide illegal incentives to inspection agencies, low-quality products are put on the market that potentially damage public health. Therefore, the regulatory authorities must allocate additional funding (*G*_*g*_) to support the stability of public health. The expected revenue of regulatory authorities is equal to (*P*_*e*_+*P*_*t*_−*C*_*r*_−*G*_*g*_). If an inspection agency refuses illegal incentives, the regulatory authority must still provide government subsidies (*S*_*t*_) to the inspection agencies. Thus, the regulatory authorities' expected revenue is equal to (*P*_*e*_−*C*_*r*_−*S*_*t*_).

When manufacturers produce high-quality ADRs, the public enjoys high-quality medical care and the government gains its trust and support as a result. In other words, the regulatory authorities can obtain social capital *E*_*g*_. The regulatory authorities provide government subsidies (*S*_*e*_) to enterprises that produce high-quality ADRs. If an inspection agency asks for illegal incentives, the regulatory authorities can impose a fine on the inspection agency and collect the fine income (*P*_*t*_). In this case, the expected revenue of the regulatory authorities is calculated as (*E*_*g*_+*P*_*t*_−*C*_*r*_−*S*_*e*_). If an inspection agency refuses the illegal incentive, the regulatory authorities must also provide government subsidies to the inspection agency (*S*_*t*_). The expected revenue for regulatory authorities is then equal to (*E*_*g*_−*C*_*r*_−*S*_*e*_−*S*_*t*_).

#### 3.4.1. Expected revenue for all parties when supervision is relaxed

##### 3.4.1.1. The expected revenue of the ADR manufacturers

Some producers, such ADR manufacturers that falsify manufacturing records or spread disinformation, promote and disseminate low-quality items, which might incur speculative costs (*V*_*e*_). If an inspection agency accepts illegal incentives, the manufacturer must then pay the illegal incentive (*K*) as well. After obtaining a false certificate issued from an inspection agency, low-quality products can be circulated in the market, and manufacturers can obtain sales revenue (*E*_*e*_). In this scenario, the manufacturer's expected revenue is calculated as (*E*_*e*_−*C*_*l*_−*V*_*e*_−*K*+*S*_*e*_). If, however, an inspection agency refuses the illegal incentive, low-quality ADRs cannot be marketed. Ultimately, the manufacturer's expected revenue is equal to (−*C*_*l*_−*V*_*e*_+*S*_*e*_).

In the presence of lax government supervision, the regulatory authorities provide government subsidies (*S*_*e*_) to manufacturers that produce high-quality products. If the ADR manufacturer produces high-quality products, there is no need to pay illegal incentives to the inspection agencies. Thus, the ADR manufacturer's expected revenue is (*E*_*e*_−*C*_*h*_+*S*_*e*_).

##### 3.4.1.2. The expected revenue of inspection agencies

In the presence of lax government supervision, the regulatory authorities will not impose fines on inspection agencies that refuse illegal incentives. If an inspection agency refuses illegal incentives, the strategic choice of ADR manufacturers will not affect the inspection agency's expected revenue. Here, the inspection agency's expected revenue is equal to (*E*_*t*_+*S*_*t*_). If an inspection agency accepts illegal incentives (*K*), it must pay the associated speculation costs (*V*_*t*_). In this scenario, regardless of whether the ADR manufacturers produce high- or low-quality products, the expected revenue of inspection agencies is equal to (*E*_*t*_+*K*−*V*_*t*_+*S*_*t*_).

##### 3.4.1.3. The expected revenue of regulatory authorities

In the presence of a lax regulatory strategy, regulatory authorities will not impose fines on manufacturers and inspection agencies. On the contrary, they will provide government subsidies to manufacturers (*S*_*e*_) and inspection agencies (*S*_*t*_). When manufacturers produce low-quality ADRs, the government cannot obtain social capital. If an inspection agency accepts illegal incentives, low-quality products can be marketed and circulated. The government must not only rectify the medical device industry but also bear the cost of social remediation (*G*_*g*_) as well as the decline in its credibility (*P*_*g*_). In this case, the regulatory authorities' expected revenue is equal to (−*G*_*g*_−*P*_*g*_−*S*_*t*_−*S*_*e*_). If an inspection agency refuses illegal incentives, low-quality ADRs cannot be marketed and circulated. Then, the regulatory authorities' expected revenue is equal to (−*S*_*t*_−*S*_*e*_). When manufacturers produce high-quality ADRs, regulatory authorities obtain the social capital (*E*_*g*_) brought about by good public health. In this scenario, the regulatory authorities' expected revenue is (*E*_*g*_−*S*_*e*_−*S*_*t*_).

According to the above assumptions and the strategic choices of the parties, the payoff matrix of the tripartite evolutionary game model can be obtained, as shown in Table 2.

**Table 2:** Payoff matrix.

	**Inspection agencies**		**Regulatory authorities**	

			**Strict supervision (z)**	**Lax supervision (1-z)**
ADR manufacturer	Produce low-quality ADRs (x)	Accepting illegal incentives (y)	*E*_*e*_−*C*_*t*_−*V*_*e*_−*K*−*P*_*e*_ *E*_*t*_−*V*_*t*_−*P*_*t*_+*K* *P*_*e*_+*P*_*t*_−*C*_*r*_−*G*_*g*_	*E*_*e*_−*C*_*t*_−*V*_*e*_−*K*−*S*_*e*_ *E*_*t*_+*K*−*V*_*t*_+*S*_*t*_ −*G*_*g*_−*P*_*g*_−*S*_*t*_−*S*_*e*_
		Refusing illegal incentives (1-y)	−*C*_*l*_−*V*_*e*_−*P*_*e*_ *E*_*t*_+*S*_*t*_ *P*_*e*_−*C*_*r*_−*S*_*t*_	−*C*_*l*_−*V*_*e*_+*S*_*e*_*E*_*t*_+*S*_*t*_−*S*_*t*_−*S*_*e*_
	Produce high-quality ADRs (1-x)	Accepting illegal incentives (y)	*E*_*e*_−*C*_*h*_+*S*_*e*_ *E*_*t*_−*V*_*t*_−*P*_*t*_ *E*_*g*_+*P*_*t*_−*C*_*r*_−*S*_*e*_	*E*_*e*_−*C*_*h*_+*S*_*e*_−*KE*_*t*_−*V*_*t*_+*S*_*t*_+*KE*_*g*_−*S*_*e*_−*S*_*t*_
		Refusing illegal incentives (1-y)	*E*_*e*_−*C*_*h*_+*S*_*e*_ *E*_*t*_+*S*_*t*_ *E*_*g*_−*C*_*r*_−*S*_*e*_−*S*_*t*_	*E*_*e*_−*C*_*h*_+*S*_*e*_*E*_*t*_+*S*_*t*_*E*_*g*_−*S*_*e*_−*S*_*t*_

### 3.5. Model solving

The expected revenue of ADR manufacturers that produce low-quality (*U*_11_) and high-quality products (*U*_12_) and their average expected revenue (Ū_1_) are as follows:


$$\begin{array}{l} U_{11}=yz\left(E_{e}-C_{l}-V_{e}-K-P_{e}\right)\\ \;+z\left(1-y\right)\left(-C_{l}-V_{e}-P_{e}\right)\\ \;+y\left(1-z\right)\left(E_{e}-C_{l}-V_{e}-K+S_{e}\right)\\ \;+\left(1-y\right)\left(1-z\right)\left(-C_{l}-V_{e}+S_{e}\right)\\ \;=-zP_{e}+yE_{e}-yK-C_{l}-V_{e}+S_{e}-zS_{e}\\ U_{12}=yz\left(E_{e}-C_{h}+S_{e}\right)+z\left(1-y\right)\left(E_{e}-C_{h}+S_{e}\right)\\ \;+y\left(1-z\right)\left(E_{e}-C_{h}+S_{e}-K\right)+\left(1-y\right)\\ \;\left(1-z\right)\left(E_{e}-C_{h}+S_{e}\right)=E_{e}-C_{h}+S_{e}-yK+yzK\\ {\bar{U}}_{1}=xU_{11}+\left(1-x\right)U_{12}. \end{array}$$

The dynamic replicator equation for the strategic selection of ADR manufacturers is:


$$\begin{array}{l} \;F\left(x\right)=\frac{dx}{dt}=x\left(U_{11}-{\bar{U}}_{1}\right)=x\left(1-x\right)\left(U_{11}-U_{12}\right)\\ =x\left(1-x\right)\left(yE_{e}-zP_{e}-C_{l}-V_{e}-zS_{e}-E_{e}+C_{h}-yzK\right). \end{array}$$

The expected revenue of inspection agencies that accept illegal incentives (*U*_21_) and refuse illegal incentives (*U*_22_) and their average expected revenue ($\bar{U_{2}}$) are as follows:


$$\begin{array}{l} \;U_{21}=xz\left(E_{t}-V_{t}-P_{t}+K\right)+x\left(1-z\right)\left(E_{t}+K-V_{t}+S_{t}\right)\\ +z\left(1-x\right)\left(E_{t}-V_{t}-P_{t}\right)+\left(1-x\right)\left(1-z\right)\left(E_{t}-V_{t}+S_{t}+K\right)\\ \;=E_{t}+K-V_{t}+S_{t}-zK-zS_{t}-zP_{t}+xzK\\ \;U_{22}=xz\left(E_{t}+S_{t}\right)+x\left(1-z\right)\left(E_{t}+S_{t}\right)\\ \;+z\left(1-x\right)\left(E_{t}+S_{t}\right)+(1-x)(1-z)\left(E_{t}+S_{t}\right)=E_{t}+S_{t}\\ \;{\bar{U}}_{2}=yU_{21}+\left(1-y\right)U_{22}. \end{array}$$

From the equation set above, the dynamic replicator equation for the inspection agencies is given by:


$$\begin{array}{l} F\left(y\right)=\frac{dy}{dt}=y\left(U_{21}-{\bar{U}}_{2}\right)=y\left(1-y\right)\left(U_{21}-U_{22}\right)\\ \;=y\left(1-y\right)\left(K-V_{t}-zK-zS_{t}-zP_{t}+xzK\right). \end{array}$$

The expected revenue of regulatory authorities that pursue strict supervision (*U*_31_), lax supervision (*U*_32_), and their average expected revenue (Ū_3_) are as follows:


$$\begin{array}{l} \;U_{31}=xy\left(P_{e}+P_{t}-C_{r}-G_{g}\right)+x\left(1-y\right)\left(P_{e}-C_{r}-S_{t}\right)\\ \;+y\left(1-x\right)\left(E_{g}+P_{t}-C_{r}-S_{e}\right)+\left(1-x\right)\left(1-y\right)\\ \;\left(E_{g}-C_{r}-S_{e}-S_{t}\right)\\ =xP_{e}-xyG_{g}+yP_{t}+E_{g}-C_{r}-S_{e}-S_{t}+yS_{t}-xE_{g}+xS_{e}\\ \;U_{32}=xy\left(-G_{g}-P_{g}-S_{t}-S_{e}\right)+x\left(1-y\right)\left(-S_{t}-S_{e}\right)\\ \;+y\left(1-x\right)\left(E_{g}-S_{e}-S_{t}\right)+\left(1-x\right)\left(1-y\right)\left(E_{g}-S_{e}-S_{t}\right)\\ \;=-xyG_{g}-xyP_{g}+E_{g}-S_{e}-S_{t}-xE_{g}\\ \;{\bar{U}}_{3}=zU_{31}+\left(1-z\right)U_{32}. \end{array}$$

The dynamic replicator equation for the regulatory authorities is as follows:


$$\begin{array}{l} F\left(z\right)=\frac{dz}{dt}=z\left(U_{31}-{\bar{U}}_{3}\right)=z\left(1-z\right)\left(U_{31}-U_{32}\right)\\ \;=z\left(1-z\right)\left(xP_{e}+yP_{t}-C_{r}+yS_{t}+xS_{e}+xyP_{g}\;\right). \end{array}$$

### 3.6. Equilibrium solution

The game model processes for regulatory authorities, ADR manufacturers, and inspection agencies are constantly evolving. That is, the probability of any strategy chosen by any of the three parties is time dependent. According to the differential equation stability principle, when all dynamic replicator equations are 0, the entire evolutionary game model will tend to stabilize (47). The equilibrium point of the tripartite evolutionary game model can be calculated by *F*(*x*) = 0, *F*(*y*) = 0, *F*(*z*) = 0 (51). That is:


$$\begin{array}{l} \;F\left(x\right)=x\left(1-x\right)\\ \left(yE_{e}-zP_{e}-C_{l}-V_{e}-zS_{e}-E_{e}+C_{h}-yzK\right)=0\\ \;F\left(y\right)=y\left(1-y\right)\\ \;\left(K-V_{t}-zK-zS_{t}-zP_{t}+xzK\right)=0\\ \;F\left(z\right)=z\left(1-z\right)(xP_{e}+yP_{t}-C_{r}\\ \;+yS_{t}+xS_{e}+xyP_{g}\;)=0. \end{array}$$

It follows that there are eight special equilibrium points: *E*_1_(0, 0, 0), *E*_2_(1, 0, 0), *E*_3_(0, 1, 0), *E*_4_(0, 0, 1), *E*_5_(1, 1, 0), *E*_6_(1, 0, 1), *E*_7_(0, 1, 1), and *E*_8_(1, 1, 1). All stakeholders adopt a pure strategy at each equilibrium point (52).

According to the dynamic replicator equations, the Jacobian matrix of the tripartite evolutionary game model can be calculated as:


$$J=\left[\begin{array}{ccc} \frac{\partial F(x)}{\partial x} & \frac{\partial F(x)}{\partial y} & \frac{\partial F(x)}{\partial z}\\ \frac{\partial F(y)}{\partial x} & \frac{\partial F(y)}{\partial y} & \frac{\partial F(y)}{\partial z}\\ \frac{\partial F(z)}{\partial x} & \frac{\partial F(z)}{\partial y} & \frac{\partial F(z)}{\partial z} \end{array}\right]=\left[\begin{array}{ccc} J_{11} & J_{12} & J_{13}\\ J_{21} & J_{22} & J_{23}\\ J_{31} & J_{32} & J_{33} \end{array}\right].$$

In the above formula:


$$\begin{array}{l} J_{11}=\left(1-2x\right)(yE_{e}-zP_{e}-C_{l}-V_{e}-zS_{e}-E_{e}+C_{h}-yzK);\\ \;J_{12}=x\left(1-x\right)\left(E_{e}-zK\right);\\ \;J_{13}=x\left(1-x\right)(-P_{e}-S_{e}-yK);\\ \;J_{21}=y\left(1-y\;\right)(zK);\\ \;J_{22}=\left(1-2y\right)\left(K-V_{t}-zK-zS_{t}-zP_{t}+xzK\right);\\ \;J_{23}=y\left(1-y\right)\left(-K-S_{t}-P_{t}+xK\right);\\ \;J_{31}=z\left(1-z\right)(P_{e}+S_{e}+yP_{g});\\ \;J_{32}=z\left(1-z\right)(P_{t}+S_{t}+xP_{g});\\ \;J_{33}=\left(1-2z\right)(xP_{e}+yP_{t}-C_{r}+yS_{t}+xS_{e}+xyP_{g}). \end{array}$$

It can be seen from Lyapunov's first method that, in the analysis of differential systems, the stability can be judged according to the positive and negative eigenvalues of the equilibrium point (53, 54). When all of the eigenvalues of the equilibrium point are negative, the point is considered an evolutionary stabilization strategy (i.e., an asymptotically stable point). When the eight pure strategy points are brought into the Jacobian matrix, the eigenvalues of the equilibrium points are obtained.

For the equilibrium point *E*_1_(0, 0, 0), the Jacobian matrix (*M*_1_) is as follows:


$$M_{1}=\left(\begin{array}{ccc} K-V_{t} & 0 & 0\\ 0 & -C_{r} & 0\\ 0 & 0 & \;C_{h}-C_{l}-E_{e}-V_{e} \end{array}\right).$$

In matrix *M*_1_, *E*_*e*_>*C*_*h*_, so it follows that *C*_*h*_−*C*_*l*_−*E*_*e*_−*V*_*e*_ <0. When *K*−*V*_*t*_ <0, the point (0, 0, 0), is the equilibrium point of evolutionary stability.

For the equilibrium point *E*_2_(1, 0, 0), the Jacobian matrix (*M*_2_) is as follows:


$$M_{2}=\left(\begin{array}{ccc} K-V_{t} & 0 & 0\\ 0 & P_{e}-C_{r}+S_{e} & 0\\ 0 & 0 & C_{l}-C_{h}+E_{e}+V_{e} \end{array}\right).$$

In matrix *M*_2_, *E*_*e*_>*C*_*h*_, so it follows that *C*_*l*_−*C*_*h*_+*E*_*e*_+*V*_*e*_>0, which implies that the necessary and sufficient conditions for the stability of the evolution of the system are not satisfied, so the point *E*_2_(1, 0, 0) is not the stable equilibrium point of the evolutionary game model.

For the equilibrium point *E*_3_(0, 1, 0), the Jacobian matrix (*M*_3_) is as follows:


$$M_{3}=\left(\begin{array}{ccc} V_{t}-K & 0 & 0\\ 0 & P_{t}-C_{r}+S_{t} & 0\\ 0 & 0 & C_{h}-C_{l}-V_{e} \end{array}\right).$$

In matrix *M*_3_, *C*_*h*_>*C*_*l*_+*K*+*V*_*e*_+*P*_*e*_+*S*_*e*_; therefore, *C*_*h*_−*C*_*l*_−*V*_*e*_>0 does not meet the necessary and sufficient conditions for the stability of the evolution of the model. It follows that the point *E*_3_(0, 1, 0) is not the stable equilibrium point of the evolutionary game model.

For the equilibrium point *E*_4_(0, 0, 1), the Jacobian matrix (*M*_4_) is as follows:


$$M_{4}=\left(\begin{array}{ccc} C_{r} & 0 & 0\\ 0 & -P_{t}-S_{t}-V_{t} & 0\\ 0 & 0 & C_{h}-C_{l}-E_{e}-P_{e}-S_{e}-V_{e} \end{array}\right).$$

In matrix *M*_4_, *C*_*r*_>0, does not meet the equilibrium conditions for the stability of the evolutionary game model. The point *E*_4_(0, 0, 1) is thus not the stable equilibrium point of the evolutionary game model.

For the equilibrium point *E*_5_(1, 1, 0), the Jacobian matrix (*M*_5_) is as follows:


$$M_{5}=\left(\begin{array}{ccc} V_{t}-K & 0 & 0\\ 0 & C_{l}-C_{h}+V_{e} & 0\\ 0 & 0 & P_{e}-C_{r}+P_{g}+P_{t}+S_{e}+S_{t} \end{array}\right).$$

In matrix *M*_5_, *C*_*h*_>*C*_*l*_+*K*+*V*_*e*_+*P*_*e*_+*S*_*e*_, so *C*_*l*_−*C*_*h*_+*V*_*e*_ <0. When *V*_*t*_−*K* <0 and *P*_*e*_−*C*_*r*_+*P*_*g*_+*P*_*t*_+*S*_*e*_+*S*_*t*_ <0, the equilibrium point *E*_5_(1, 1, 0) satisfies the necessary and sufficient conditions for the stability of the evolutionary game model, which may be the stable equilibrium point of the evolutionary game model.

For the equilibrium point *E*_6_(1, 0, 1), the Jacobian matrix (*M*_6_) is as follows:


$$\begin{array}{l} M_{6}\\ =\left(\begin{array}{ccc} C_{r}-P_{e}-S_{e} & 0 & 0\\ 0 & K-P_{t}-S_{t}-V_{t} & 0\\ 0 & 0 & C_{l}-C_{h}+E_{e}+P_{e}+S_{e}+V_{e} \end{array}\right). \end{array}$$

In matrix *M*_6_, *E*_*e*_>*C*_*h*_, so *C*_*l*_−*C*_*h*_+*E*_*e*_+*P*_*e*_+*S*_*e*_+*V*_*e*_>0, and the conditions for the stability of the evolutionary game model are not satisfied. Thus, the point *E*_6_(1, 0, 1) is not the stable equilibrium point of the evolutionary game model.

For the equilibrium point *E*_7_(0, 1, 1), the Jacobian matrix (*M*_7_) is as follows:


$$\begin{array}{l} M_{7}\\ \;=\text{​}\left(\begin{array}{ccc} \text{​}C_{r}-P_{t}-S_{t} & 0 & 0\\ 0 & P_{t}+S_{t}+V_{t} & 0\\ 0 & 0 & C_{h}-C_{l}-K-P_{e}-S_{e}-V_{e}\text{​​} \end{array}\right)\text{​}. \end{array}$$

In matrix *M*_7_, *P*_*t*_+*S*_*t*_+*V*_*t*_>0 and the conditions for the stability of the evolutionary game model are not met, so the point *E*_7_(0, 1, 1) is not the stable equilibrium point of the evolutionary game model.

For the equilibrium point *E*_8_(1, 1, 1), the Jacobian matrix (*M*_8_) is as follows:


$$M_{8}\;=\left(\begin{array}{ccc} P_{t}-K+S_{t}+V_{t} & 0 & 0\\ 0 & C_{r}-P_{e}-P_{g}-P_{t}-S_{e}-S_{t} & 0\\ 0 & 0 & C_{l}-C_{h}+K+P_{e}+S_{e}+V_{e} \end{array}\right).$$

In matrix *M*_8_, *C*_*h*_>*C*_*l*_+*K*+*V*_*e*_+*P*_*e*_+*S*_*e*_, so *C*_*l*_−*C*_*h*_+*K*+*P*_*e*_+*S*_*e*_+*V*_*e*_ <0. When *P*_*t*_−*K*+*S*_*t*_+*V*_*t*_ <0 and *C*_*r*_−*P*_*e*_−*P*_*g*_−*P*_*t*_−*S*_*e*_−*S*_*t*_ <0, the equilibrium point *E*_8_(1, 1, 1) satisfies the necessary and sufficient conditions for the evolutionary game model, which may be inferred as its stable equilibrium point.

We now combine the conditions of the relative size of the parameters assumed above. Judging from the condition that the eigenvalue is negative, points *E*_2_, *E*_3_, *E*_4_, *E*_6_ and *E*_7_ are not evolutionary stability points. A further analysis shows the conditions for the above three equilibrium points (*E*_1_, *E*_5_, *E*_8_) to become evolutionary stability points, and the positive and negative conditions of the eigenvalues are shown in the following Table 3.

**Table 3:** Evolutionary stability conditions for equilibrium points.

**Equilibrium point**	**Evolutionary stabilization conditions**
*E*_1_(0, 0, 0)	*K*−*V*_*t*_ <0
*E*_5_(1, 1, 0)	*V*_*t*_−*K* <0 *P*_*e*_−*C*_*r*_+*P*_*g*_+*P*_*t*_+*S*_*e*_+*S*_*t*_ <0
*E*_8_(1, 1, 1)	*V*_*t*_−*K*+*S*_*t*_+*P*_*t*_ <0 *C*_*r*_−*P*_*e*_−*P*_*g*_−*P*_*t*_−*S*_*e*_−*S*_*t*_ <0

As can be seen from the above table, there can be three evolutionarily stable equilibrium points in a tripartite evolutionary game model. Among them, *E*_1_(0, 0, 0) indicates that manufacturers produce high-quality ADRs, inspection agencies refuse illegal incentives, and the supervision is lax. When the evolutionary game model remains stable at that point, it is conducive to maximizing the wellbeing of society. However, under certain conditions, the stable equilibrium point of the evolutionary game model may evolve into point (1, 1, 1).

## 4. Results

Three evolutionary stabilization strategies are obtained by calculating and analyzing the model. In order to more intuitively observe the evolution trajectory of each stakeholder and their parameter sensitivity, this paper uses MATLAB 2021a to simulate the evolutionary stabilization strategies and the related parameters.

### 4.1. Simulation of evolutionary stabilization strategies

**Figure 2:**
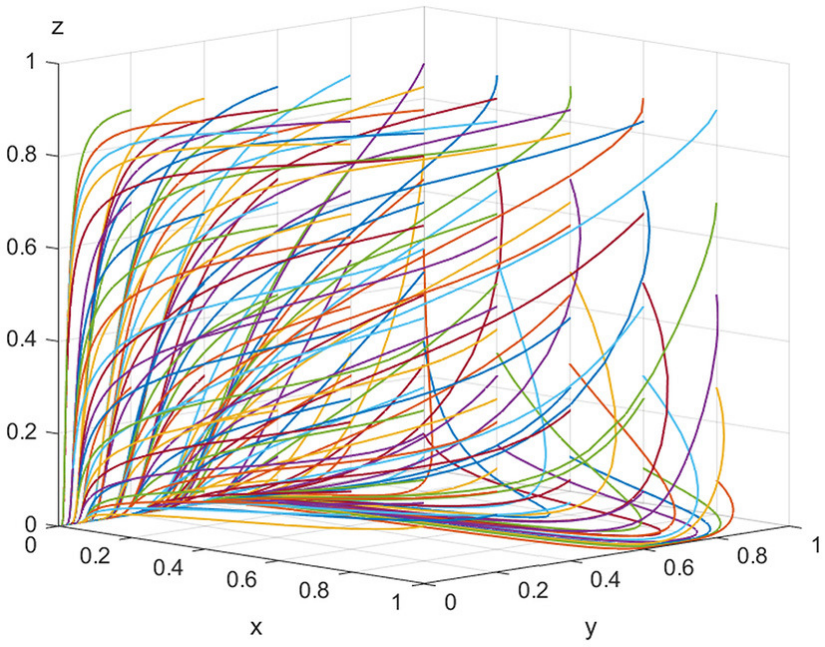
Equilibrium point (0, 0, 0) parameter simulation.

For the equilibrium point (0, 0, 0), when *K*<*V*_*t*_, *C*_*h*_−*C*_*l*_−*E*_*e*_−*V*_*e*_ <0 is satisfied, the point (0, 0, 0) is the evolutionarily stable equilibrium point. In order to satisfy the above conditions, it is assumed that *E*_*e*_ = 500, *C*_*h*_ = 180, *C*_*l*_ = 30, *V*_*e*_ = 20, *K* = 30, *P*_*e*_ = 10, *S*_*e*_ = 15, *V*_*t*_ = 40,*P*_*t*_ = 30,*S*_*t*_ = 20, *C*_*r*_ = 120, *and P*_*g*_ = 20. As shown in Figure 2, when the speculative cost (*V*_*t*_) faced by inspection agencies is higher than the illegal incentive amount (*K*) offered by ADR manufacturers, the final evolutionary result is the same equilibrium point (0, 0, 0) regardless of the manufacturers' and regulatory authorities' initial strategy. Inspection agencies get bribes that are less than their speculative costs, therefore they are compelled to reject bribes from ADR manufacturers. Regulatory authorities are also not required to incur regulatory costs to regulate ADR manufacturers and inspection agencies. In other words, lawmakers must strengthen the severity of penalties for inspection agencies in the legal requirements in order to prevent excessive enforcement expenses for law enforcement and ADR manufacturers that create substandard goods.

**Figure 3:**
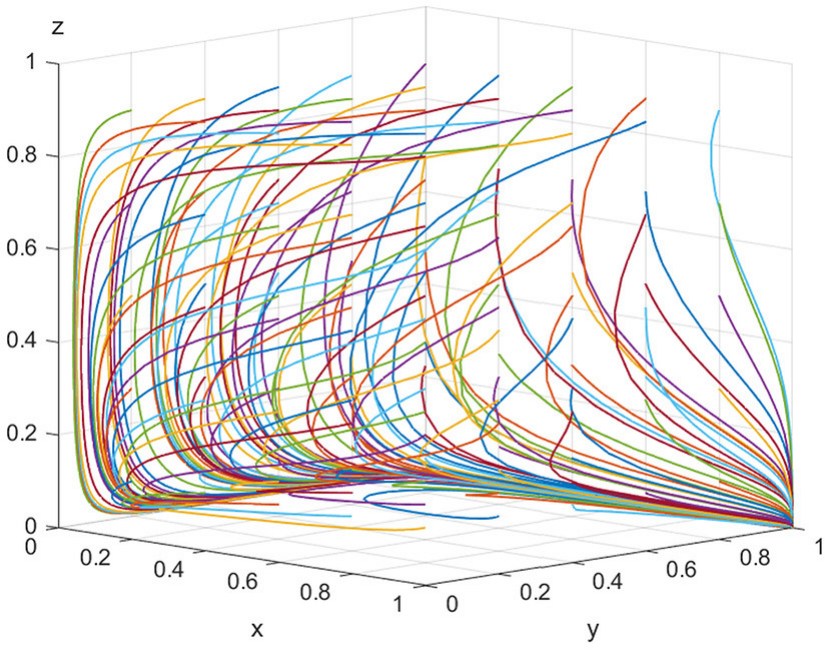
Equilibrium point (1, 1, 0) parameter simulation.

For equilibrium point (1, 1, 0), when the conditions *K*>*V*_*t*_ and *C*_*r*_>*P*_*e*_+*P*_*g*_+*P*_*t*_+*S*_*e*_+*S*_*t*_ are satisfied, the point (1, 1, 0) is the stable equilibrium point of the evolutionary game model. To satisfy the above conditions, it is assumed that *E*_*e*_ = 500, *C*_*h*_ = 180, *C*_*l*_ = 30, *V*_*e*_ = 20, *K* = 100, *P*_*e*_ = 10, *S*_*e*_ = 15, *V*_*t*_ = 40, *P*_*t*_ = 30, *S*_*t*_ = 20, *C*_*r*_ = 120, *and P*_*g*_ = 20. As shown in Figure 3, as evolution progresses, the equilibrium point gradually shifts from point (0, 0, 0) to point (1, 1, 0). In other words, if the illegal incentives paid by ADR manufacturers exceed the inspection agencies' speculative costs, the possibility of illegal activity occurring between them rises. However, the regulatory authorities still prefer lax supervision. Thus, for all parties in the model, the optimal strategies are “produce low-quality ADRs,” “accept illegal incentives,” and “choose lax supervision,” respectively.

**Figure 4:**
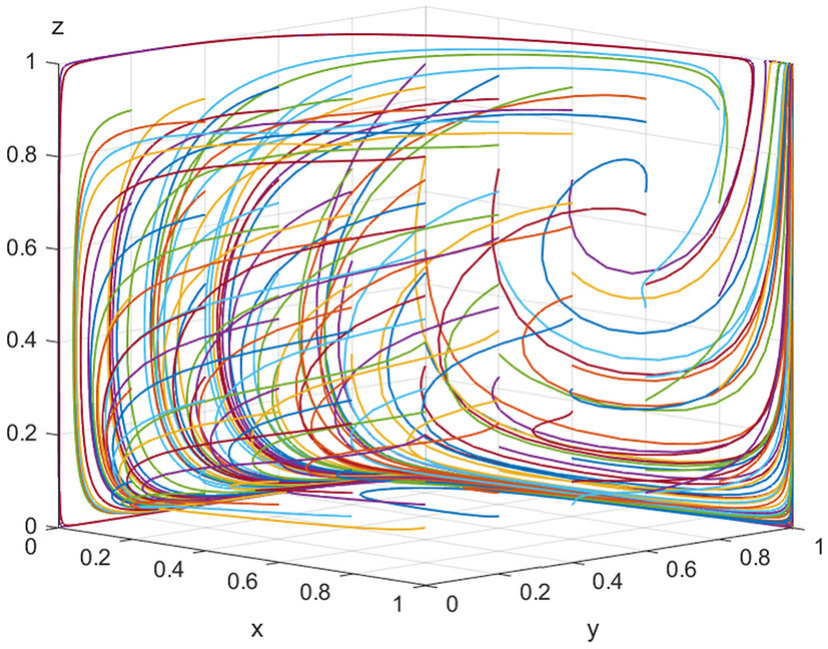
Equilibrium point (1, 1, 0) parameter simulation.

For equilibrium point(1, 1, 1), when *K*>*V*_*t*_+*S*_*t*_+*P*_*t*_ and *C*_*r*_<*P*_*e*_+*P*_*g*_+*P*_*t*_+*S*_*e*_+*S*_*t*_ are satisfied, the point (1, 1, 1) is the stable equilibrium point of the evolutionary game model. To satisfy the above conditions, we assume that *E*_*e*_ = 500, *C*_*h*_ = 180, *C*_*l*_ = 30, *V*_*e*_ = 20, *K* = 100, *P*_*e*_ = 10, *S*_*e*_ = 15, *V*_*t*_ = 40, *P*_*t*_ = 30, *S*_*t*_ = 20, *C*_*r*_ = 120, *and P*_*g*_ = 100. As shown in Figure 4, with the increase in the illegal incentives offered by ADR manufacturers and the government's loss of credibility, the possibility of inspection agencies accepting illegal incentives also increases and the government choosing “strict supervision” tends to 100%. This shows that inspection agencies will accept illegal incentives when they are sufficiently high. If low-quality ADRs are released to the market in large quantities, public health is seriously threatened and the government's credibility will be severely impaired. Regulatory authorities will be forced to take strict regulatory measures. Finally, for all parties in the model, the optimal strategies are “produce low-quality ADRs,” “accept illegal incentives,” and “choose strict supervision,” respectively.

Figures 3, 4 show that when the monetary amount of illegal incentives is fixed, the loss of government credibility will be a key factor in determining the regulatory authorities' optimal decisions. In other words, if there is no public supervision of the government, the regulatory authorities will inevitably choose to relax supervision resulting in the market being flooded with a high quantity of low-quality ADRs, resulting in a potentially serious threat to public health.

### 4.2. Parameter analysis

The equilibrium point (0, 0, 0) is the optimal choice to maximize social welfare. At this point, the optimal strategic combination of the three parties is to produce high-quality ADRs, refuse illegal incentives, and choose lax supervision. It is intuitive that producing high-quality ADRs can better protect public health and choosing lax supervision can reduce regulatory costs. The refusal of inspection agencies to engage in rent-seeking can encourage manufacturers to invest more resources in producing high-quality ADRs. However, under certain conditions, the equilibrium points (0, 0, 0), (1, 1, 0) and (1, 1, 1) may shift to each other. When the model is at equilibrium points (1, 1, 0) and (1, 1, 1), the overall wellbeing of society is not improved. Therefore, this paper performs a sensitivity analysis of certain key parameters in the three possible equilibrium points to better reveal the influencing factors in each party's strategic choices. These key parameters include the number of illegal incentives (*K*), the inspection agencies' speculative cost (*V*_*t*_), the regulatory authorities' regulatory cost (*C*_*r*_), and the loss of government credibility (*P*_*g*_).

#### 4.2.1. Sensitivity analysis of illegal incentives

**Figure 5:**
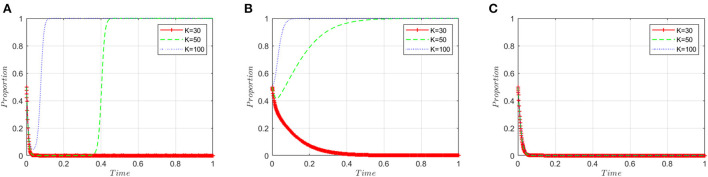
Sensitivity analysis of illegal incentives. **(A)** ADR manufacturers. **(B)** Inspection agencies. **(C)** Regulatory authorities.

This section analyzes the stakeholders' sensitivity to the illegal incentives (*K*) offered by manufacturers. Based on the simulated value of the equilibrium point (0, 0, 0), the illegal incentive amount (*K*) is set to 30, 50, and 100, respectively. Figure 5A shows that when the illegal incentives are low, manufacturers tend to produce high-quality ADRs. However, when the illegal incentives exceed a certain threshold, the probability of manufacturers choosing to produce low-quality ADRs first decreases and then increases in an inverted “U” shape, and finally converges on the “produce low-quality ADRs” strategy. Figure 5B shows that when the illegal incentives are low, inspection agencies choose to reject illegal incentives. When the illegal incentives exceed the threshold, inspection agencies will choose to accept them. Figure 5C shows that the three curves almost overlap and the level of illegal incentives has little effect on the policy choices of regulatory authorities.

#### 4.2.2. Sensitivity analysis of regulatory costs

**Figure 6:**
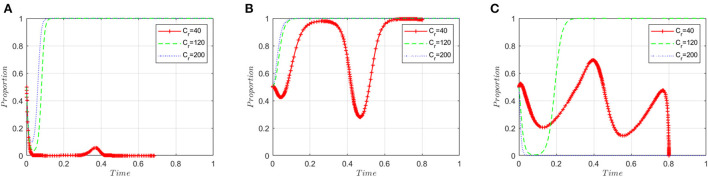
Sensitivity analysis of regulatory costs. **(A)** ADR manufacturers. **(B)** Inspection agencies. **(C)** Regulatory authorities.

This section analyzes stakeholders' sensitivity to regulatory costs (*C*_*r*_). On the basis of the simulation values at the equilibrium point (1, 1, 1), the regulatory costs (*C*_*r*_) are set to 40, 120 and 200, respectively. Figure 6A shows that when the regulatory costs are low, manufacturers are more tightly regulated and therefore more inclined to produce high-quality ADRs. However, with the rising cost of supervision, regulatory authorities must loosen their supervision such that manufacturers change their preference to producing low-quality ADRs.

Figure 6B shows that inspection agencies are more likely to accept illegal incentives as regulatory costs increase. When the cost of supervision is low, inspection agencies will be strictly supervised by government regulatory institutions, which will result in the probability of accepting illegal incentives initially decreasing but then rapidly increasing.

Figure 6C shows that when regulatory costs are low, it is easy for regulatory authorities to detect and stop the wrongdoing of ADR manufacturers and inspection agencies, so that ADR manufacturers choose to produce high-quality ADRs and inspection agencies choose to refuse bribes. The evolutionary game system will eventually converge to the equilibrium point (0, 0, 0), and the regulatory authorities will choose a lax regulatory strategy. As regulatory costs rise, the regulatory authorities first adopt a strict regulatory strategy before adopting a lax regulatory strategy. When the cost of regulation is too high, regulatory authorities are unable to supervise and have to choose a lax regulatory strategy.

#### 4.2.3. Sensitivity analysis of the loss of government credibility

**Figure 7:**
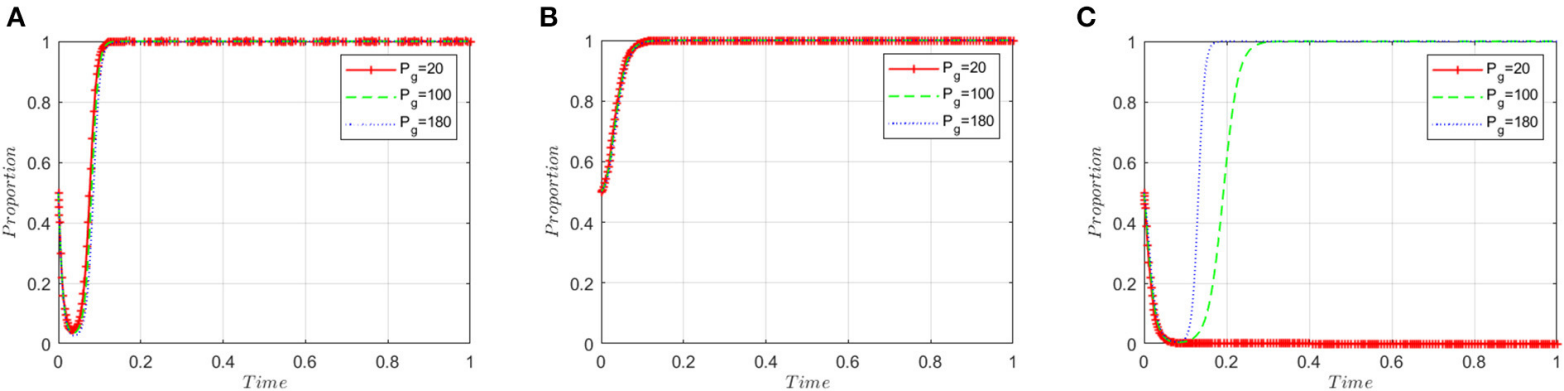
Sensitivity analysis of the loss of government credibility. **(A)** ADR manufacturers. **(B)** Inspection agencies. **(C)** Regulatory authorities.

This section analyzes stakeholders' sensitivity to the loss of government credibility (*P*_*g*_). On the basis of the simulated value of the equilibrium point (1, 1, 1), the government's credibility loss (*P*_*g*_) is set to 20, 100, and 180, respectively. Figure 7A shows that the loss of government credibility has little impact on the decision-making of ADR manufacturers; Figure 7B shows that it has little effect on the decision-making of inspection agencies; Figure 7C shows that when the loss of government credibility is small, regulatory authorities are more likely to choose a lax regulatory strategy. When the loss of credibility exceeds the threshold, government regulators will change their strategy. As the loss of government credibility increases, the convergence speed of the government choosing the “strict supervision” strategy will accelerate. This means an increase in the government's loss of credibility will prompt it to accelerate its adoption of strict regulatory strategies.

## 5. Discussion

The significance of investigating ADR production and circulation became increasingly evident as we dealt with the practical challenges brought on by the COVID-19 pandemic. This paper examines the process of ADR production, certification, and marketing and further attempts to construct an evolutionary game model to consider each stakeholder's optimal strategy. The point (0, 0, 0) represents the optimal evolutionary stabilization strategy, which is the most likely to promote social welfare. Under this tripartite evolutionary game model scenario, manufacturers produce high-quality ADRs, inspection agencies refuse illegal incentives, and regulatory agencies exercise proper, non-invasive supervision.

First, legislators should integrate ADRs into the drug price monitoring system (DPMS) and restrict the number of illicit incentives (*K*). Illegal bribes are financed by product earnings. The greater the product's profit margin, the greater the number of bribes given to inspection agencies by ADR manufacturers, and the greater the possibility of successful bribery (55). As demonstrated in Figure 5B, in the face of considerable financial temptation, inspection agencies will eventually accept illegal incentives, resulting in the proliferation of low-quality ADRs on the market.

Article 62 of the Law of the People's Republic of China on Basic Medical and Health Care and the Promotion of Health (hereinafter Basic Health Law) stipulates that the state establishes and improves a drug price monitoring system, conducts investigations into prices, strengthens drug price supervision, and provides inspections. The DPMS envisaged by Article 62, however, does not apply to medical devices. The government does not regulate the prices of medical products, resulting in price manipulation, price fraud, and unfair competition. Clinical trials, medical research, patent applications, and marketing authorization for medical devices are governed by the Civil Code, the Biosafety Law, the Patent Law, and the Physicians Law, respectively. These laws continue to exclude the DPMS for medical devices. The government does not have the legal jurisdiction to monitor ADR prices. Therefore, China's legislators should amend the law to include medical devices, such as ADR, in the existing DPMS to combat price manipulation, price fraud, and unfair competition and promote industry-wide competition.

Second, the government should improve law enforcement and reduce regulatory costs (*C*_*r*_). In practice, it is extremely challenging for the government to regulate the research, production, operation, and use of medical devices, as well as the companies and individuals involved. Information asymmetries in the medical device industry are quite severe, creating regulatory gaps. Article 75 of the Medical Devices Regulation stipulates that the regulatory authorities in the law enforcement assigned to inspect medical devices must delegate qualified medical device inspection agencies and bear the costs of inspection. The inspection cost has certainly raised regulatory expenses (*C*_*r*_), impeding the authorities' ability to enforce the law effectively.

If regulatory authorities deregulate ADR manufacturers due to exorbitant regulatory costs, low-quality ADR will become a threat to public health. If ADR manufacturers and inspection agencies are found to be in breach of the law, they should ultimately be responsible for the inspection costs associated with law enforcement by regulatory authorities. In addition, regulatory authorities can utilize blockchain technology to construct a traceable network for monitoring clinical trials, medical research, patent applications, and marketing authorization of ADRs, thereby minimizing the expense of constant monitoring.

Third, the government should safeguard the people's right to freedom of expression and prevent a loss of government credibility (*P*_*g*_). People's right to freedom of expression is not properly guaranteed in China (56). A preference for a lax regulatory strategy that allows low-quality ADRs to circulate on the market has resulted from the absence of effective public oversight and the low level of government credibility incurred by regulatory authorities.

Article 62 of the Medical Devices Regulation gives individuals the right to report adverse incidents associated with medical devices to regulatory authorities or inspection agencies. However, the Medical Devices Regulation does not establish the regulatory authority's responsibilities upon receipt of a citizen's report. In other words, regulatory authorities may disregard adverse incidents discovered by citizens involving medical devices. Internal government oversight finds it challenging to alter the strategy of regulatory authorities. Public criticism from citizens is a significant factor in convincing regulators to implement stringent policies. The Chinese government should build an online information platform for reporting adverse events associated with medical devices and permit citizens to submit queries. Faced with a further loss of government credibility, the regulatory authorities will need to implement a stringent regulatory policy to halt the production and registration of low-quality ADRs.

## 6. Conclusion

Considering the possible rent-seeking behaviors of ADR manufacturers and inspection agencies, this paper constructs a tripartite evolutionary game model that includes ADR manufacturers, inspection agencies, and regulatory authorities. We analyze the stability of the optimal strategy choice of each party, the stability of the equilibrium strategy combination of the game system, and the influence relationship of each element, and verify the validity of the results through simulation analysis. However, there are still certain shortcomings in this research. Different methodologies can be tested. For example, taking the perspective of a legal case study to explore how to improve the punishment for violations of the law in the ADR industry. The effectiveness of different strategies can be analyzed through either qualitative or quantitative methods. Besides, accepting bribes is common in developing countries. However, it is less common in developed countries. It means that the model in this paper may not be valid in developed countries. Nevertheless, this paper's findings could still provide helpful insights for many developing countries regulating the product quality of ADR.

## Data availability statement

The original contributions presented in the study are included in the article/supplementary material, further inquiries can be directed to the corresponding authors.

## Author contributions

ZH and BC conducted the modeling and data analysis and drafted of the manuscript. ZH, ZF, and XW conceptualized the study, designed the experiment, and contributed to the manuscript. ZH, ZF, and BC contributed to the acquisition and computation of data. All authors critically revised the manuscript for important intellectual content and approved the final version.
